# Responsiveness of the EQ-5D in breast cancer patients in their first year after treatment

**DOI:** 10.1186/1477-7525-7-11

**Published:** 2009-02-07

**Authors:** Merel L Kimman, Carmen D Dirksen, Philippe Lambin, Liesbeth J Boersma

**Affiliations:** 1 MAASTRO Clinic, School for Oncology and Developmental Biology (GROW), Maastricht University Medical Centre, Maastricht, the Netherlands; 2 Department of Radiation Oncology (MAASTRO), Maastricht University Medical Centre, Maastricht, the Netherlands; 3 Department of Clinical Epidemiology and Medical Technology Assessment, Maastricht University Medical Centre, Maastricht, the Netherlands

## Abstract

**Background/aim:**

The EQ-5D is a generic health-related quality of life (HRQoL) measure that is used for the purpose of economic evaluations of health interventions. Therefore, it has to be responsive to meaningful changes in health in the patient population under investigation. The aim of this study was to investigate the responsiveness of the EQ-5D in breast cancer patients in their first year after treatment.

**Methods:**

The subscale global health of the disease-specific HRQoL measure EORTC QLQ-C30 was used as a reference instrument to determine meaningful changes in health and identify subgroups of patients: patients reporting a moderate-large deterioration, small deterioration, a small improvement, moderate-large improvement, or no change in health status. Responsiveness was evaluated by calculating standardized response means (SRMs) in the five subgroups of patients and performing analysis of variance procedures. The two HRQoL measures were filled out two weeks and one year after finalizing curative treatment for breast cancer (n = 192).

**Results:**

The EQ-5D was able to capture both improvements and deteriorations in HRQoL. SRMs of the EQ VAS and EQ-5D Index were close to zero in the subgroup reporting no change and increased and decreased adequately in the subgroups reporting small and moderate changes. Additional analysis of variance procedures showed that the EQ-5D was able to differentiate between subgroups of patients with no change and moderate-large deterioration or improvement in health.

**Conclusion:**

The EQ-5D seems an appropriate measure for the purpose of economic evaluations of health intervention in breast cancer patients after treatment.

**Trial registration:**

Current Controlled Trials ISRCTN74071417.

## Introduction

With an estimated 1.15 million new cases worldwide each year and a relatively good prognosis, breast cancer is the most prevalent cancer in the world today [1]. After curative treatment for breast cancer, women attend frequent follow-up visits to be examined for possible local or regional recurrence or a second primary breast tumor, and to receive psychosocial support [2,3]. However, no strong evidence exists that regular follow-up is effective with regard to disease free survival or overall survival [4-6], or in providing psychosocial support [7,8]. Hence, the assessment of outcomes like patient satisfaction and health-related quality of life (HRQoL) is common practice in clinical oncology trials investigating alternative follow-up strategies and psychosocial interventions for breast cancer survivors [9-18]. Given the high prevalence of breast cancer and budget constraints in health care, it is also important to understand the impact of alternative strategies on economic outcomes. Therefore, clinical trials are increasingly incorporating generic HRQoL measures, such as the EQ-5D, for the purpose of economic evaluations [19]. The EQ-5D is a standardized multi-dimensional health state classification system. It generates a single index score for each health state [20]. Index scores, in turn, can be used to calculate quality adjusted life years (QALYs), which is the most preferred summary outcome measure in economic evaluations [21].

A substantial and growing body of literature regarding the usefulness of the EQ-5D in cancer has emerged, supporting its validity and reliability [19]. However, the responsiveness of the EQ-5D, defined as its ability to capture true underlying changes in the patients' health status over time [22], is highly dependent on patient population and setting. In comparison with disease-specific instruments, the responsiveness of the EQ-5D was found to be comparable in one study [23], but more often it is found to be less responsive than disease-specific instruments [24-27]. Hence, the usefulness of the EQ-5D may be limited if it is not able to detect changes in health status in the patient population under investigation.

To our knowledge, the responsiveness of the EQ-5D has not yet been examined in breast cancer patients after treatment. Therefore, we use data from a randomized clinical trial investigating several follow-up strategies for curatively treated breast cancer patients [10] to address whether the EQ-5D is responsive to changes in HRQoL in a population of breast cancer patients in their first year after treatment.

## Methodology

### Study population

Participants were enrolled in a randomized clinical trial investigating the cost-effectiveness of nurse-led telephone follow-up and a short educational group program after curative treatment for breast cancer (MaCare trial, ISRCTN 74071417) [10]. Patients in the trial were all female, treated for breast cancer with curative intent, and had no concomitant tumors or comorbidity requiring hospital visits. There were no age restrictions. Patients were included in the trial after finalizing treatment and after giving written informed consent. Treatment included surgery and/or radiotherapy and/or chemotherapy. Follow-up appointments took place at three, six, nine and twelve months after treatment. For the purpose of studying the responsiveness of the EQ-5D, patients who had had their twelve months follow-up were eligible. The EQ-5D and the disease-specific EORTC QLQ-C30 were sent to patients at home two weeks after the end of treatment (T0) and twelve months after treatment (T1). Of 220 eligible patients, 29 patients failed to complete both instruments at both measurements due to either random missings within the instruments (n = 19) or because they were a study drop-out (n = 10). A total of 192 patients were therefore included in the analysis. Their demographic and clinical characteristics can be found in table 1. Patients were analyzed regardless of follow-up strategy in the trial.

**Table 1:** Characteristics of participants (n = 192)

**Characteristic**	**Descriptive statistic**
Age	
Mean (SD)	55.8 (10.1) yrs
Range	23–79 yrs
Level of education	
Low	69 (35.9%)
Middle	96 (50.0%)
High	27 (14.1%)
Tumor stage	
Stage I	99 (51.6%)
Stage II	61 (31.8%)
Stage III	17 (8.6%)
Unknown	15 (7.8%)
Treatment modality	
Surgery	17 (8.9%)
Surgery and radiotherapy	107 (55.7%)
Surgery and chemotherapy	13 (6.8%)
Surgery and radiotherapy and chemotherapy	55 (28.6%)
Hormonal therapy	65 (34%)

The MaCare trial was approved by the Independent Ethics Committee of MAASTRO Clinic.

### HRQoL Instruments

#### EQ-5D

The EQ-5D is a short generic health-related quality of life instrument that consists of two parts: a self-classifier and a Visual Analogue Scale (EQ VAS). The self-classifier comprises five items relating to problems in the following domains: mobility, self-care, usual activities, pain/discomfort and anxiety/depression [20]. Each domain has three levels, namely, "no problems", "some problems" and "severe problems". Combinations of these categories define a total of 243 health states. Dolan et al [28] have presented 42 of these health states to approximately 3000 members of a representative sample of the UK general population, which were valued using the time-trade-off (TTO) technique. Based on these valuations, for each health state a utility score can be deducted, called the EQ-5D Index score. These EQ-5D Index scores may vary between -0.59 (worst health) and 1.00 (perfect health). On the EQ VAS respondents can indicate their overall self-perceived health state on a scale ranging from 0 to 100, where 0 is equivalent to the worst imaginable health state and 100 is equivalent to the best imaginable health state.

#### EORTC QLQ-C30

The EORTC QLQ-C30 from the European Organization for Research and Treatment of Cancer is a self-administered disease-specific HRQoL questionnaire and is validated for oncology clinical research [29-31]. It has also been validated [32] and found to be responsive [33] specifically in breast cancer patients and is widely used in breast cancer research investigating HRQoL after treatment [34-40]. The HRQoL questionnaire consists of 30 items. After transformation, the EORTC QLQ-C30 has several multi-item functional subscales (e.g. physical, emotional functioning), multi-item symptom scales (e.g. fatigue, pain), a global health subscale, and single items to assess symptoms (e.g. sleep disturbance). Scores on the functional and global health scales range from 0 to 100, where a higher scale score represents a higher level of functioning and therefore HRQoL.

### Analyses of responsiveness

To assess the responsiveness of the EQ-5D three steps were taken, following recommendations recently published by Revicki et al (2008). First, a criterion, or anchor, that is related to the measure under investigation, was selected to identify whether patients had changed (either improved or worsened) over time. Second, when the relationship between the anchor and EQ-5D was confirmed, patients were classified into subgroups according to changes in their health status. Third, to examine responsiveness, statistical indicators for change were calculated and analysis of variance procedures were performed.

#### Step 1: Selecting an anchor; global health of the EORTC QLQ-C30

Selecting anchors should be based on criteria of relevance for the disease indication, clinical acceptance and validity, and evidence that the anchors have some relationship with the measure under investigation [41]. For this study, the subscale global health of the EORTC QLQ-C30 was proposed as a criterion for clinical change. The global health subscale consists of two items: (1) How would you rate your physical condition during the past week? and; (2) How would you rate your overall quality of life during the past week?

Correlations between global health scores and the EQ-5D Index and EQ VAS were calculated to examine whether the anchor was acceptable [41]. It is recommended that 0.30–0.35 is used as a correlation threshold to define acceptable association between an anchor and a change score on the HRQoL outcome measure [41].

#### Step 2: Classifying patients into subgroups

Change scores on global health of the EORTC QLQ-C30 were used to identify subgroups of patients. In an analysis of the clinical significance of changes in HRQoL, Osoba et al (1994) showed that patients judge a change between 5–10 on the global health scale of the EORTC QLQ-C30 to be small, between 10–20 to be moderate, and more than 20 to be large [33,42]. Consequently, a change smaller than 5 points was considered to be no change. Taking into account both deteriorations and improvements, this results in a maximum of 7 subgroups.

#### Step 3: Examining responsiveness

Responsiveness to change was evaluated using a statistical indicator, the standardized response mean (SRM). The SRM is the change in score divided by the standard deviation of the change in score. It is independent of sample size and widely used today [43]. SRMs were calculated for the EQ-5D Index and EQ VAS, for all subgroups of patients. Scores were interpreted using benchmarks for effect sizes: 0.20 through 0.49 was interpreted as small, 0.50 through 0.79 as moderate and ≥ 0.80 as large [44]. Additionally, analysis of variance, with Games Howell post hoc procedures, was performed to compare the mean change scores on the EQ-5D Index and EQ VAS between the 'no change' subgroup and the other subgroups identified in step 2.

## Results

### Step 1. Selecting an anchor

The global health scale of the EORTC QLQ-C30 correlated to the change scores of the EQ-5D Index and EQ VAS (r = 0.423 and r = 0.634 respectively). Hence, global health was found to be an appropriate anchor and was used to classify subgroups.

### Step 2. Classifying patients into subgroups

After twelve months, 6 patients (3%) reported a large deterioration on global health, 17 (9%) reported a moderate deterioration, 14 (7%) reported a small deterioration, 55 (28%) reported no change, 28 (16%) reported a small improvement, 32 (17%) a moderate improvement and 40 (21%) reported a large improvement on global health.

Due to a relatively small number of patients reporting a moderate or large deterioration, it was decided to create one subgroup for patients with both moderate and large deteriorations ('moderate-large deterioration') and, for easy comparison, also one subgroup for both moderate and large improvements ('moderate-large improvement'). Hence, five subgroups were identified, classifying patients reporting a (1) moderate-large deterioration (n = 23), (2) small deterioration (n = 14), (3) no change (n = 55), (4) a small improvement (n = 28) and (5) moderate-large improvement in health status (n = 72).

### Step 3. Examining responsiveness

Mean baseline scores, scores at the twelve month measurement and change scores are presented for all HRQoL measures in table 2. The EQ VAS and EQ-5D Index both moved in the expected direction, indicating negative changes (deterioration) in the subgroups reporting deterioration on global health of the EORTC QLQ-C30 and positive changes (improvements) in the subgroups reporting improvements on global health. Accordingly, only a minor change on the EQ VAS and no change on the EQ-5D Index were reported in the no change subgroup of the EORTC QLQ-C30.

**Table 2:** Baseline scores (T0), twelve months scores (T1) and mean change scores with standard deviations.

	**EORTC QLQ-C30 global health**			**EQ VAS**				**EQ 5D Index**			
Subgroup	T0	T1	Δ (sd)	T0	T1	Δ (sd)	**SRM**	T0	T1	Δ (sd)	**SRM**

Moderate-large deterioration (n = 23)	79.3	56.9	-22.5 (10.8)	73.0	59.8	-13.2 (11.2)	**-1.17**	0.72	0.57	-0.15 (0.29)	**-0.52**

Small deterioration (n = 14)	75.6	67.3	-8.3 (0.0)	74.4	69.4	-5.1 (12.0)	**-0.42**	0.73	0.72	-0.01 (0.18)	**-0.05**

No change (n = 55)	80.9	80.9	0.0 (0.0)	79.0	79.9	0.7 (8.8)	**0.08**	0.82	0.82	0.00 (0.21)	**0.01**

Small improvement (n = 28)	71.2	80.1	8.3 (0.0)	70.9	77.7	6.1 (7.7)	**0.79**	0.78	0.80	0.02 (0.14)	**0.16**

Moderate-large improvement (n = 72)	58.2	85.6	27.4 (11.9)	65.0	77.4	12.1 (12.7)	**0.95**	0.71	0.83	0.13 (0.20)	**0.62**

SRMs of the EQ VAS and EQ-5D Index for all subgroups of patients.

To examine responsiveness, SRMs were calculated for the EQ-5D Index and EQ VAS (table 2). In the subgroup of patients whose global health had not changed, accordingly, neither the SRM of the EQ-5D Index, nor of the EQ VAS indicated an effect. SRMs of the EQ-5D Index for the subgroups indicating a small deterioration or small improvement were too small (i.e. SRM < 0.20) to be considered as an effect. In contrast, SRMs of the EQ VAS indicated a small effect in these subgroups. SRMs of the subgroups with moderate and large improvements or deteriorations in global health indicated a moderate effect on the EQ-5D Index (i.e. SRM > 0.50) and a large effect on the EQ VAS (i.e. SRM > 0.80).

Analysis of variance procedures were performed to evaluate whether the EQ-5D could discriminate between the five subgroups (table 3). Results indicated that when the EQ-5D Index score was used as the outcome measure, the subgroup reporting no change on global health differed significantly from the subgroup reporting moderate and large improvements. The subgroups reporting small improvements or a small or moderate and large deterioration could not be differentiated from the 'no change' subgroup. The EQ VAS on the other hand was able to discriminate between the 'no change' subgroup and the subgroups reporting a moderate and large improvement and moderate and large deterioration.

**Table 3:** Analysis of variance

**Global health EORTC QLQ-C30**		**EQ-5D Index**		**EQ VAS**	
	Subgroup	Mean difference (SE)	p-value	Mean difference (SE)	p-value

	Moderate-large deterioration (n = 23)	-0.14 (0.07)	.228	-13.88 (2.65)*	.000
No change	Small deterioration (n = 14)	0.01 (0.05)	1.000	-5.78 (3.44)	.470
(n = 55)	Small improvement (n = 28)	0.02 (0.04)	.984	5.37 (1.93)	.054
	Moderate-large improvement (n = 72)	0.13 (0.04)*	0.006	11.38 (1.97)*	.000

* Subgroups are significantly different at a 0.05 significance level

## Discussion

An increasing number of clinical trials is investigating the effectiveness of follow-up strategies and psychosocial interventions for breast cancer patients after treatment, using HRQoL as an important outcome measure [45,46]. Hence, a good responsiveness of the HRQoL measure used seems essential. Our study showed that the EQ-5D was able to detect both improvements and deteriorations in health. However, according to Cohen's benchmarks for effect sizes [44], the EQ-5D Index was not responsive to small changes in health. The inability of the EQ-5D Index to detect small changes might be explained by its structure. It is generally acknowledged that more response options lead to a higher responsiveness [26]. The domains of the EQ-5D have only three response levels, making it difficult to pick up small changes in health. In addition, in the subgroup of patients reporting no change and the subgroup reporting a small improvement on global health, baseline scores on the EQ-5D Index were relatively high. These high scores were a result of large proportions of respondents already in the top category of domains of the EQ-5D. This ceiling effect is a well known feature of the EQ-5D and left little room for improvement [47]. A straightforward solution would be to attempt to produce a better, more responsive, generic index measure. Recent studies on an EQ-5D with five response levels for each domain showed increased descriptive power and suggest better discriminatory power [48,49]. Hence a less severe ceiling effect and increased benefit in the detection of small health changes are expected [49]. Unfortunately, an official five-level descriptive system is not yet available.

Additional analysis of variance procedures to investigate responsiveness showed that the EQ-5D Index and the EQ VAS both could not differentiate between subgroups reporting no change and small changes in global health. For the EQ-5D Index this was in accordance with the small SRMs in these subgroups. For the EQ VAS however, the non-significant differences were unexpected, as the SRMs indicated moderate effects. This inability of the EQ VAS to discriminate might be explained by the small number of patients in these subgroups (n = 14 and n = 28 respectively). Analysis of variance procedures and especially post hoc procedures are sensitive to population variances and differences in sample size in subgroups. Hence, with a larger sample size, the EQ VAS might have been able to differentiate between subgroups with no change and small changes in health. This argument also holds true for the EQ-5D Index, which could not discriminate between the 'no change' subgroup and the subgroup reporting a moderate-large deterioration in health (n = 23).

A limitation of this study was that the responsiveness was investigated using a single anchor, while ideally multiple anchors should be used to investigate the responsiveness of an instrument [50]. A clinical variable, such as whether or not a recurrence was detected, would be a suitable second anchor to classify subgroups of patients. However, in the clinical trial from which participants were used for these analyses, only few (< 10) recurrences were reported, and unfortunately, these participants were study drop-outs. Hence, an appropriate second anchor was not available. Further research into the responsiveness of the EQ-5D in breast cancer patients should aim to include multiple anchors.

In summary, results of this study showed that the EQ-5D was able to capture both improvements and deteriorations in HRQoL of breast cancer patients after treatment, but small changes in health were not recognized as being meaningful. However, in economic evaluations the EQ-5D is primarily used to measure outcome for QALY analysis rather than measuring HRQoL for clinical purposes. Within the framework of economic evaluations, an incremental cost-effectiveness ratio (i.e. additional cost per QALY gained) is more informative than the difference in HRQoL alone. Therefore, a small difference in the EQ-5D Index might still be meaningful when additional costs for such a change in HRQoL are very low. Hence, the EQ-5D should indeed be able to pick up relevant changes in health and should be able to differentiate between subgroups of patients to some extent, but cut-off points for effect sizes or discriminative ability are less relevant in the context of economic evaluations.

## Conclusion

In this study the responsiveness of the EQ-5D was investigated for its use in economic evaluations of health interventions in breast cancer patients after primary treatment. The EQ-5D was able to detect improvements and deteriorations in health and could discriminate between patients with no change in health and patients with moderate-large changes in health. Therefore, the EQ-5D seems an appropriate HRQoL measure for economic evaluations in breast cancer patients after treatment.

## Competing interests

The authors declare that they have no competing interests.

## Authors' contributions

MK was responsible for the data collection and drafted the manuscript. MK works under direct supervision of CD and LB. PL, CD and LB read and corrected draft versions of the manuscript.

## References

[B1] Parkin DM, Bray F, Ferlay J, Pisani P (2005). Global cancer statistics, 2002. CA Cancer J Clin.

[B2] ESMO (2001). ESMO Minimum Clinical Recommendations for diagnosis, adjuvant treatment and follow-up of primary breast cancer. Ann Oncol.

[B3] Khatcheressian JL, Wolff AC, Smith TJ, Grunfeld E, Muss HB, Vogel VG, Halberg F, Somerfield MR, Davidson NE (2006). American Society of Clinical Oncology 2006 update of the breast cancer follow-up and management guidelines in the adjuvant setting. J Clin Oncol.

[B4] de Bock GH, Bonnema J, Hage J van der, Kievit J, Velde CJ van de (2004). Effectiveness of routine visits and routine tests in detecting isolated locoregional recurrences after treatment for early-stage invasive breast cancer: a meta-analysis and systematic review. J Clin Oncol.

[B5] Jacobs HJ, van Dijck JA, de Kleijn EM, Kiemeney LA, Verbeek AL (2001). Routine follow-up examinations in breast cancer patients have minimal impact on life expectancy: a simulation study. Ann Oncol.

[B6] te Boekhorst DS, Peer NG, Sluis RF van der, Wobbes T, Ruers TJ (2001). Periodic follow-up after breast cancer and the effect on survival. Eur J Surg.

[B7] Allen A (2002). The meaning of the breast cancer follow-up experience for the women who attend. Eur J Oncol Nurs.

[B8] Pennery E, Mallet J (2000). A preliminary study of patients' perceptions of routine follow-up after treatment for breast cancer. Eur J Oncol Nurs.

[B9] Grunfeld E, Gray A, Mant D, Yudkin P, Adewuyi-Dalton R, Coyle D, Cole D, Stewart J, Fitzpatrick R, Vessey M (1999). Follow-up of breast cancer in primary care vs specialist care: results of an economic evaluation. Br J Cancer.

[B10] Kimman ML, Voogd AC, Dirksen CD, Falger P, Hupperets P, Keymeulen K, Hebly M, Dehing C, Lambin P, Boersma LJ (2007). Improving the quality and efficiency of follow-up after curative treatment for breast cancer – rationale and study design of the MaCare trial. BMC Cancer.

[B11] Kimman ML, Voogd AC, Dirksen CD, Falger P, Hupperets P, Keymeulen K, Hebly M, Dehing C, Lambin P, Boersma LJ (2007). Follow-up after curative treatment for breast cancer: why do we still adhere to frequent outpatient clinic visits?. Eur J Cancer.

[B12] Koinberg IL, Fridlund B, Engholm GB, Holmberg L (2004). Nurse-led follow-up on demand or by a physician after breast cancer surgery: a randomised study. Eur J Oncol Nurs.

[B13] Antoni MH, Lehman JM, Kilbourn KM, Boyers AE, Culver JL, Alferi SM, Yount SE, McGregor BA, Arena PL, Harris SD, Price AA, Carver CS (2001). Cognitive-behavioral stress management intervention decreases the prevalence of depression and enhances benefit finding among women under treatment for early-stage breast cancer. Health Psychol.

[B14] Grunfeld E, Levine MN, Julian JA, Coyle D, Szechtman B, Mirsky D, Verma S, Dent S, Sawka C, Pritchard KI, Ginsburg D, Wood M, Whelan T (2006). Randomized trial of long-term follow-up for early-stage breast cancer: a comparison of family physician versus specialist care. J Clin Oncol.

[B15] Helgeson VS, Cohen S, Schulz R, Yasko J (2001). Long-term effects of educational and peer discussion group interventions on adjustment to breast cancer. Health Psychol.

[B16] Marcus AC, Garrett KM, Cella D, Wenzel LB, Brady MJ, Crane LA, McClatchey MW, Kluhsman BC, Pate-Willig M (1998). Telephone counseling of breast cancer patients after treatment: a description of a randomized clinical trial. Psychooncology.

[B17] Meneses KD, McNees P, Loerzel VW, Su X, Zhang Y, Hassey LA (2007). Transition from treatment to survivorship: effects of a psychoeducational intervention on quality of life in breast cancer survivors. Oncology nursing forum.

[B18] Sandgren AK, McCaul KD (2003). Short-term effects of telephone therapy for breast cancer patients. Health Psychol.

[B19] Pickard AS, Wilke CT, Lin H-W, Lloyd A (2007). Health utilities using the EQ-5D in studies of cancer. PharmacoEconomics.

[B20] Group E (1990). EuroQol – a new facility for the measurement of health-related quality of life. The EuroQol Group. Health Policy.

[B21] Drummond MF, Sculpher MJ, Torrance GW, O'Brien BJ, Stoddart GL (2005). Methods for the economic evaluation of health care programmes.

[B22] Terwee CB, Dekker FW, Wiersinga WM, Prummel MF, Bossuyt PM (2003). On assessing responsiveness of health-related quality of life instruments: guidelines for instrument evaluation. Qual Life Res.

[B23] Krabbe PF, Peerenboom L, Langenhoff BS, Ruers TJ (2004). Responsiveness of the generic EQ-5D summary measure compared to the disease-specific EORTC QLQ C-30. Qual Life Res.

[B24] Eurich DT, Johnson JA, Reid KJ, Spertus JA (2006). Assessing responsiveness of generic and specific health related quality of life measures in heart failure. Health Qual Life Outcomes.

[B25] Willige G van de, Wiersma D, Nienhuis FJ, Jenner JA (2005). Changes in quality of life in chronic psychiatric patients: a comparison between EuroQol (EQ-5D) and WHOQoL. Qual Life Res.

[B26] Wiebe S, Guyatt G, Weaver B, Matijevic S, Sidwell C (2003). Comparative responsiveness of generic and specific quality-of-life instruments. J Clin Epidemiol.

[B27] Krahn M, Bremner KE, Tomlinson G, Ritvo P, Irvine J, Naglie G (2007). Responsiveness of disease-specific and generic utility instruments in prostate cancer patients. Qual Life Res.

[B28] Dolan P (1997). Modeling valuations for EuroQol health states. Med Care.

[B29] Aaronson NK, Ahmedzai S, Bergman B, Bullinger M, Cull A, Duez NJ, Filiberti A, Flechtner H, Fleishman SB, de Haes JC (1993). The European Organization for Research and Treatment of Cancer QLQ-C30: a quality-of-life instrument for use in international clinical trials in oncology. J Natl Cancer Inst.

[B30] Bottomley A, Aaronson NK (2007). International perspective on health-related quality-of-life research in cancer clinical trials: the European Organisation for Research and Treatment of Cancer experience. J Clin Oncol.

[B31] Fayers P, Bottomley A (2002). Quality of life research within the EORTC-the EORTC QLQ-C30. European Organisation for Research and Treatment of Cancer. Eur J Cancer.

[B32] McLachlan SA, Devins GM, Goodwin PJ (1998). Validation of the European Organization for Research and Treatment of Cancer Quality of Life Questionnaire (QLQ-C30) as a measure of psychosocial function in breast cancer patients. Eur J Cancer.

[B33] Osoba D, Zee B, Pater J, Warr D, Kaizer L, Latreille J (1994). Psychometric properties and responsiveness of the EORTC quality of Life Questionnaire (QLQ-C30) in patients with breast, ovarian and lung cancer. Quality of life research.

[B34] Arndt V, Merx H, Stegmaier C, Ziegler H, Brenner H (2005). Persistence of restrictions in quality of life from the first to the third year after diagnosis in women with breast cancer. J Clin Oncol.

[B35] Arndt V, Merx H, Sturmer T, Stegmaier C, Ziegler H, Brenner H (2004). Age-specific detriments to quality of life among breast cancer patients one year after diagnosis. Eur J Cancer.

[B36] Arndt V, Stegmaier C, Ziegler H, Brenner H (2006). A population-based study of the impact of specific symptoms on quality of life in women with breast cancer 1 year after diagnosis. Cancer.

[B37] Goodwin PJ, Ennis M, Bordeleau LJ, Pritchard KI, Trudeau ME, Koo J, Hood N (2004). Health-related quality of life and psychosocial status in breast cancer prognosis: analysis of multiple variables. J Clin Oncol.

[B38] Helgesson O, Lissner L, Mansson J, Bengtsson C (2007). Quality of life in cancer survivors as observed in a population study of Swedish women. Scand J Prim Health Care.

[B39] Schou I, Ekeberg O, Sandvik L, Hjermstad MJ, Ruland CM (2005). Multiple predictors of health-related quality of life in early stage breast cancer. Data from a year follow-up study compared with the general population. Qual Life Res.

[B40] Waldmann A, Pritzkuleit R, Raspe H, Katalinic A (2007). The OVIS study: health related quality of life measured by the EORTC QLQ-C30 and -BR23 in German female patients with breast cancer from Schleswig-Holstein. Qual Life Res.

[B41] Revicki D, Hays RD, Cella D, Sloan J (2008). Recommended methods for determining responsiveness and minimally important differences for patient-reported outcomes. Journal of clinical epidemiology.

[B42] Osoba D, Rodrigues G, Myles J, Zee B, Pater J (1998). Interpreting the significance of changes in health-related quality-of-life scores. J Clin Oncol.

[B43] Husted JA, Cook RJ, Farewell VT, Gladman DD (2000). Methods for assessing responsiveness: a critical review and recommendations. J Clin Epidemiol.

[B44] Cohen J (1988). Statistical Power Analysis for Behavioral Science.

[B45] Goodwin PJ, Black JT, Bordeleau LJ, Ganz PA (2003). Health-related quality-of-life measurement in randomized clinical trials in breast cancer – taking stock. Journal of the National Cancer Institute.

[B46] Montazeri A (2008). Health-related quality of life in breast cancer patients: a bibliographic review of the literature from 1974 to 2007. Journal of experimental & clinical cancer research.

[B47] Brazier J, Roberts J, Tsuchiya A, Busschbach J (2004). A comparison of the EQ-5D and SF-6D across seven patient groups. Health Econ.

[B48] Janssen MF, Birnie E, Bonsel GJ (2008). Quantification of the level descriptors for the standard EQ-5D three-level system and a five-level version according to two methods. Qual Life Res.

[B49] Janssen MF, Birnie E, Haagsma JA, Bonsel GJ (2008). Comparing the standard EQ-5D three-level system with a five-level version. Value Health.

[B50] Guyatt GH, Osoba D, Wu AW, Wyrwich KW, Norman GR (2002). Methods to explain the clinical significance of health status measures. Mayo Clinic proceedings.

